# The Immediate Effect of Dry Needling Electric Muscle Stimulation on the Position of Atlas

**DOI:** 10.3390/jcm13144097

**Published:** 2024-07-13

**Authors:** Rob Sillevis, Daniel Cerdeira, Jared Yankovich, Anne Weller Hansen

**Affiliations:** Department of Rehabilitation Sciences, Marieb College of Health and Human Services, Florida Gulf Coast University, Fort Myers, FL 33965, USA; dacerdeira6124@eagle.fgcu.edu (D.C.); jaredfyankovich@gmail.com (J.Y.); awhansen@fgcu.edu (A.W.H.)

**Keywords:** atlas, cervicogenic headache, dry needling, electrotherapy, oblique capitis inferior muscle

## Abstract

**Background**: cervicogenic headaches are common and are believed to be the cause of dysfunction in the upper cervical spine. The mobility and the position of the atlas have been identified as a cause of upper cervical dysfunction. The mobility of the atlas is entirely under the control of the suboccipital muscles. The oblique capitis inferior muscle has a rotatory effect on the atlas when contracted. This study evaluated the immediate effects of a dry needling electrical stimulation-induced contraction of the left oblique capitis inferior muscle on the position and mobility of the atlas in the atlantoaxial joint. **Methods**: thirty-one subjects participated in this within-subject repeated measure study design. Each subject underwent a pre-measures neck flexion rotation test, palpation of the atlas position, and measurement of the length of the right oblique capitis inferior muscle with musculoskeletal ultrasound imaging. The pre-measures were repeated after two five-second tetanic contractions of the oblique capitis inferior muscle. **Results**: post-intervention analysis revealed significant changes in the length of the right oblique capitis inferior muscle. This length change correlated with the palpated positional default position immediately after the intervention. **Conclusions**: two five-second tetanic contractions of the left oblique capitis inferior muscle immediately affected the position of the atlas in the atlantoaxial joint. In our subjects, 90% displayed a positional default in the left rotation, and this was correlated with a change in the neck flexion rotation test. This study supports the notion that suboccipital muscle tonicity can result in mobility dysfunction and, thus, conditions such as cervicogenic headaches.

## 1. Introduction

Musculoskeletal (MSK) disorders are the leading source of pain and disability globally and carry a significant economic burden [1]. In 2015, over 264 million lost workdays were due to chronic neck and back pain. The estimated annual financial burden of MSK-related disorders in the United States is $874 billion [1]. Neck and back pain disorders are the most common cause of disability amongst adults in prime working ages 18 to 64 years [1]. Despite the high prevalence and economic burden, the care provided to patients with MSK disorders is highly variable and regularly has suboptimal outcomes.

An example of a cervical MSK disorder is a cervicogenic headache (CGH). CGHs are prevalent in 20% of those who suffer from chronic headaches, and it can become a debilitating condition [2,3,4]. CGHs are present in 54.3% of people with both traumatic and nontraumatic neck injuries. The International Headache Society defines CGHs as “headaches caused by a disorder of the cervical spine and its components such as bone, disc, and soft tissue elements, usually but not invariably accompanied by referred neck pain” [5]. CGHs are believed to be caused by improper mechanical function of the upper cervical spine, which leads to stiffness, pain, and the onset of headache symptoms [5]. 

Understanding the pathogenesis of a CGH is essential in successfully treating the underlying cause in the cervical spine and neck. Causative mechanisms behind CGHs are believed to be a combination of neurological and mechanical dysfunctions [3,6,7]. Limited rotational motion between the atlas and axis is commonly found in subjects with CGH [8]. Additionally, unilateral hypertonicity of the oblique capitis inferior muscle (OCI) is frequently present in patients with CGHs [7,9]. The suboccipital muscles stabilize the upper cervical spine by controlling motion between the occiput, atlas, and axis [9]. Uncontrolled muscle hypertonicity within the suboccipital muscles may contribute to a decreased segmental motion and possibly disturb the normal anatomical alignment of the upper cervical spine [10]. Poor upright positioning of the upper cervical spine has not only been related to CGH, but also to conditions such as cervical myofascial pain syndrome, cervical degenerative disc disease, and cervical tension headaches [11,12,13].

The dura mater is the outermost layer of the meningeal layers that envelops and protects the brain and spinal cord [14]. The dura mater appears to be a sensitive structure, and irritation contributes to the development of headaches [14]. The dura mater in the upper cervical spine receives innervation from the branches of the trigeminal, vagus, and first three cervical nerves (C1–C3) [15]. The meningeal layer of the dura anchors to the vertebral column through the myovertebral ligaments and likely prevents excessive motion of the spinal cord [7]. Additionally, Sillevis and Hogg [10] identified ligaments anchoring the meningeal layer to structures along the cervical spine, such as the nuchal ligament and the suboccipital muscles [16,17,18,19]. The clinical meaning of these collagenous connections between the dura, vertebrae, nuchal ligament, and suboccipital muscles remains elusive. However, the position of the atlas, or suboccipital muscle tonicity, might contribute to the development of CGHs [13,15].

Increased muscle tone in the suboccipital muscles is commonly found clinically in subjects with CGH. One manual treatment approach targeting muscle tone is the use of an isometric static contraction [20,21]. Static contractions activate tension-sensitive interneurons and produce a reflexive relaxation of the agonist muscle, inhibiting overall muscle tone [20,21]. This was demonstrated in the upper cervical spine following an isometric contraction of the OCI, leading to an immediate increase in the length of the contralateral OCI identified using musculoskeletal ultrasound (MSK US) [22]. Additionally, it was shown that isometric contractions can alter vertebral anatomical positional relationships [20]. As it relates to the upper cervical spine, MSK US allows for the reliable and real-time assessment of the muscle thickness and cross-sectional area of the suboccipital muscles [3,23,24]. 

Of all the suboccipital muscles, the OCI is the only muscle that attaches to the atlas and axis. Therefore, it has been proposed that unilateral OCI contractions will produce a homolateral rotational position of the atlas relative to the axis [3]. This positional default has been previously reported in subjects with CGHs [8]. Musculoskeletal ultrasound imaging can effectively be used to introduce a needle in the anatomical region that is being observed [25]. It has been demonstrated that ultrasound-guided needling is more accurate than regional palpation for needle placement [25]. Dry needling is the insertion of thin monofilament needles, without medication, directly into muscles, ligaments, tendons, or fascia to manage neuromusculoskeletal conditions [26]. 

This study aimed to evaluate whether tetanic contractions of the left OCI caused by direct dry needle electrical stimulation result in an immediate change in the contralateral OCI length measured using MSK US and the positional relationship between the atlas and axis measured using palpation in a group of healthy subjects. The hypothesis for this study was that electrical stimulation of the left OCI muscle would immediately change the position of the atlas.

## 2. Material and Methods

### 2.1. Subjects

This quasi-experimental study used a method of convenience sampling with a within-subject repeated measure design. G*power, version 3.1, was used to perform an a priori power analysis assuming a normal distribution of data in combination with a power of 0.80, alpha of 0.5, a medium effect size of 0.50, and three measurements. The G*power 3.1 *t*-test, with a mean difference from the constant, indicated that the minimum number of required subjects was 34. However, only 31 subjects were recruited over four weeks to participate in this within-group study design. To be included in this study, subjects had to be between the ages of 18 and 65 and be able to read English so that proper written consent could be given. Exclusion criteria included needle phobia, open wounds, lymphedema, cellulitis or other skin infections, malignancy, or blood-clotting disorders. This study received approval in April 2023 from the Institutional Review Board (IRB) of Florida Gulf Coast University (#2023-19). All subjects provided written consent prior to participating in the study. 

### 2.2. Study Protocol

Prior to collecting direct measurements, quantitative data, including the subject’s age, sex, history of neck pain, and history of headaches, were collected. Next, the subject was placed supine, and the atlantoaxial mobility was assessed via the flexion–rotation test (FRT) by investigators one and two. During the FRT, the cervical spine is placed in a maximum forward bend position. In this position, the cervical facet joint capsules of C7-T1 through C2–C3 are assumed to be maximally stretched to the end range. If, at that point, rotation of the head is carried out, this would represent pure atlantoaxial rotation. The promotion assessment was carried out in both directions and measured using a standard with a goniometer [3]. Reliability of the FRT in patients with cervicogenic headaches has been demonstrated by Hall et al. [27]. The FRT has a high sensitivity (90–91%), specificity (88–90%), and overall diagnostic accuracy (91%) [27]. 

**Figure 1:**
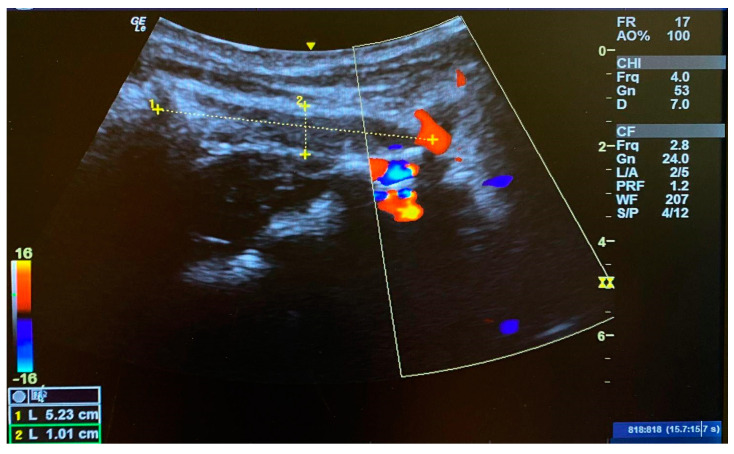
Measurement of the length of the right OCI. The measurement is taken from the spinous process of C2 to the right transverse process of the atlas. The reference point laterally is the right vertebral artery.

**Figure 2:**
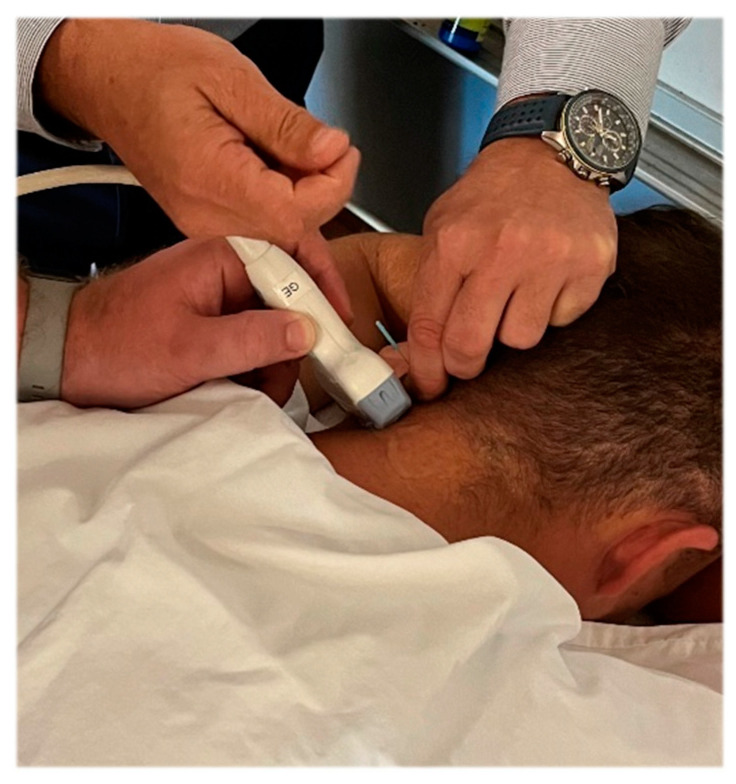
Needle placement while using MSK US for localization of the left OCI. Due to hairline issues, the MSK US was positioned below the needle entry points so the anatomical structures were visualized in the MSK US image.

**Figure 3:**
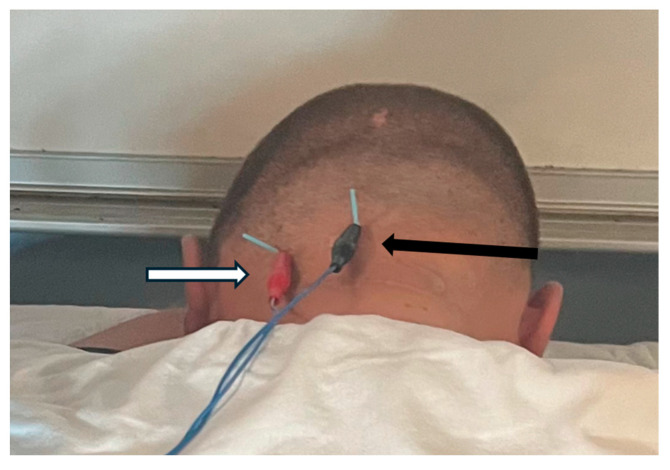
The black arrow (right side) represents the location of the spinous process of the axis, and the white arrow (left side) represents the left transverse process of the atlas. Attached to each dry needle is an electrode.

Investigator three manually palpated the bilateral transverse processes of the atlas while each subject was seated erect and looking forward. In this position, the atlas position was identified as either a neutral, right, or left rotation in each subject. The subject remained seated, and the neck was fully flexed. While in this position, investigator two palpated each subject’s right OCI for MSK US probe placement and used imaging to measure the diameter of the right OCI muscle (Figure 1). The distance between the spinous process of C2 and the transverse process of C1 was also measured and served as a secondary objective rotational positional indicator. Sillevis and Swanick [3] previously described this method. After these measures, investigator three used ultrasound imaging to guide two dry needles. To reach the OCI muscle under the trapezius and semispinalis muscles, dry needles with a length of 40 mm were used (Figure 2). Once the dry needles were inserted into the left OCI muscle, they were connected to an electrical stimulation electrotherapy (ESE) unit (Figure 3). The pulse duration was set at 400 µs with a frequency of 30 Hz to obtain a five-second tetanic contraction. The amplitude was gradually increased until a tetanic contraction of the left OCI muscle was visible under the musculoskeletal imaging field. This contraction was maintained for five seconds and repeated once more. Following the induced contractions, the dry needles were removed, and investigator two immediately remeasured the distance between the spinous process of C2 from the transverse process of C1 and the diameter of the right OCI muscle using musculoskeletal ultrasound. After the subject was placed upright, investigator three palpated the transverse processes of the atlas again to identify either a neutral, right, or left rotation position. Then, the subject was placed supine, and investigators one and two remeasured the subject’s FRT.

### 2.3. Data Analysis

Data analysis was conducted using the Statistical Package for Social Science (SPSS) version 28. All data were analyzed using a confidence interval of 95% and a significance level of 0.05. The data were analyzed for normal distribution using the Shapiro–Wilk test of normality, and all data were normally distributed with *p* > 0.05; for that reason, the assumption for parametric statistics was satisfied. The cervical range of motion was measured in degrees utilizing a goniometer. The diameter of the OCI muscle and the distance between the spinous process of C2 and the transverse process of C1 were measured in millimeters with the internal measurement option of the MSK US device. Descriptive statistics were used to analyze the total study sample. The *t*-test was used to determine the difference in OCI muscle length and the difference in distance between the spinous process of C2 and the transverse process of C1, an indicator of the atlas position. To further determine the effect size, Cohen’s d was used. Pearson’s correlation was used to determine if there was a correlation between the FRT and the direct observational measure using MSK US. 

## 3. Results

All 31 subjects completed the measurement protocol. The mean age of the subjects was 26.31, ranging from 22 to 45. Fourteen subjects were male, and 90% of the subjects were right-handed. Of the subject sample, 18.8% were prone sleepers, 28.1% were supine sleepers, and most of the subjects were side sleepers (53.1%). The mean FRT before intervention was 39.19 degrees for left rotation and 39 degrees for right rotation. The pre-intervention length of the left OCI was 4.09 cm. 

### 3.1. The Position of the Atlas Is Measured with Atlas Transverse Process Palpation

**Figure 4:**
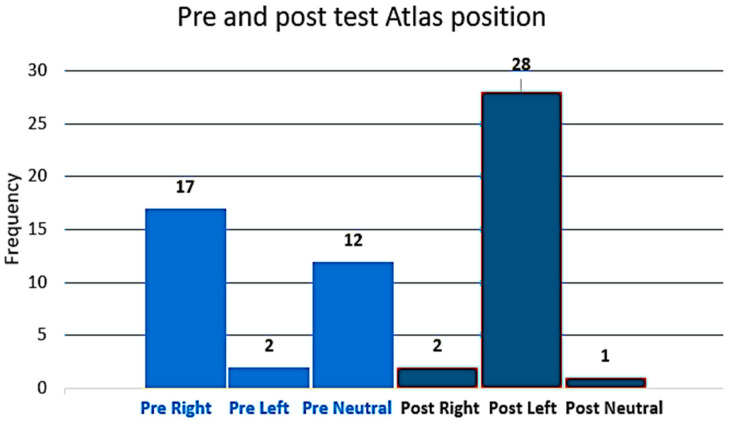
Pre and post intervention palpation for right, left, and neutral atlas positions. Frequency represents the number of subjects.

The pre-intervention positional relationship of the atlas revealed that 54.8% of the subjects were in a right rotation, 6% were in a left rotation, and 39% were identified as having a neutral rotation (Figure 4). There was a significant shift (*p* > 0.001) in the positional relationship of the atlas. Following the electrical stimulation of the left OCI, 90% of the subjects (N = 28) were identified as having an atlas in the left rotated position, 6% (N = 2) were in the right rotated position, and only one subject (4%) was in the neutral position. The results support the notion that palpation for the position of the atlas can identify an altered position of the atlas immediately following a muscle contraction of the OCI induced using direct dry needle electric stimulation. 

### 3.2. Muscle Length of the OCI Pre- and Post-Intervention

MSK US was used to determine the length of the OCI in the seated FRT neutral position. The spinous process of the axis and the transverse process of the atlas transverse process can be used as landmarks to obtain an impression of the OCI position [3]. The mean length of the right OCI was 4.064 cm before contraction of the left OCI. Post contraction of the left OCI, the mean length of the right OCI was 4.087 cm, indicating an overall increase in muscle length. The *t*-test reveals that the pre- and post-contraction difference is significant with *p* < 0.01 (Table 1). The effect size, measured using Cohen’s d, was d = 0.51, indicating a medium effect. The results demonstrate that immediately following two tetanic muscle contractions of the left OCI induced using direct dry needle electric stimulation, the length of the right OCI significantly increased, which supports the notion of a left rotation of the atlas.

### 3.3. Correlation between Post-Intervention Muscle Length Test and FRT

The Pearson’s correlation test was used to identify a relationship between the post-intervention muscle length measure and the FRT. A statistically significant (*p* = 0.02) inverse relationship exists between the post-intervention muscle length measure and the right rotation from the FRT measure (Table 2). Although there appears to be a moderate relationship between the left rotation from the FRT measure and the post-intervention muscle length, this is not significant (*p* = 0.84). The results demonstrate that following two tetanic contractions of the left OCI, there is a decreased right rotation of the atlas during the FRT. A left rotation of the atlas due to increased muscle tone of the left OCI might account for the decrease in right rotation of the atlas.

## 4. Discussion

This study aimed to evaluate whether two five-second tetanic contractions of the left OCI muscle contraction caused by direct intra-muscular electrical dry-needling stimulation would change the contralateral OCI muscle length, the positional relationship between the atlas and axis, and FRT measurements. This was based on the belief that the upper cervical spine segments contribute to common dysfunctions such as CGHs [4]. Previously, the relationship between the suboccipital muscles and the dura has been demonstrated [10,17,28]. Sillevis and Hogg [10] identified collagenous connections between the suboccipital muscles, the nuchal ligament, and the cervical dura. These connections strongly imply that muscle tone, bone position, and or joint capsules could play a significant role in musculoskeletal pathology related to the upper cervical spine syndrome [29]. This concurs with the findings of Kahkeshani et al. [30], who reported that the myodural bridge from the dura to the suboccipital muscles likely contributes to the pathogenesis of CGHs.

The suboccipital muscles play a pivotal role in controlling the biomechanical movement of the upper cervical spine [31]. Based on their direct attachments, the rectus capitis major (RCM) and the OCI are well positioned to control the rotational motion of the atlas [32]. Both muscles are active during normal neck rotation. During this motion, the head will turn the neck, allowing the atlas to turn on the axis [31]. The first 45 degrees of rotational movement of the atlas on the axis is not directly limited by any passive anatomical structures. This lack of passive restriction implies that the atlas can relatively freely rotate around the dens of the axis [29]. Although the transverse ligament maintains the position of the atlas in an anterior and posterior direction on the dens, it has not been found that this restricts the rotation of the atlas in the first 45 degrees on either side [10]. Despite the atlas’s lack of passive control, there is muscular control by the suboccipital muscles, which initiate and guide movement in the upper cervical spine. If hypertonicity of the suboccipital muscles is present, it can be hypothesized that this would reduce upper cervical mobility. Especially, OCI and RCM hypertonicity would have a negative effect on the rotational movement ability of the atlas relative to the axis. The results of this study support this hypothesis, as increased muscle tone of the left OCI was correlated to decreased atlantoaxial rotational mobility. This is clinically relevant as reduced atlantoaxial joint mobility has been related to pathologies such as myofascial pain syndrome, tension type headaches, and CGH [12]. Guo et al. [33] demonstrated that a decrease in suboccipital muscle tone was correlated with a decrease in myofascial symptoms in the neck. Hammerle et al. [34] also identified that the pain pressure threshold in the suboccipital muscles correlated to CGHs.

The hypothesis for this study was that muscle contraction of an isolated left OCI would result in an immediate left rotation of the atlas. This hypothesis is supported by similar observations from Forbes [9], who suggested that hypertonicity of the suboccipital muscles may change the position of the atlas. Additionally, Chen et al. [7] identified that the thickness of the OCI measured with MRI was smaller on the painful side when compared to the non-painful side in patients with CGHs. Although Chen et al. [7] did not report directly on the position of the atlas in the atlantoaxial joint, one can assume that asymmetrical muscle thickness should lead to a rotational change of the atlas toward the thicker OCI muscle. This hypothesis is further supported by the results of this study, which demonstrated that two five-second tetanic contractions of the left OCI resulted in an immediate change in the position of the atlas for 90% of the subjects. Originally, Mulligan introduced the “positional default” to describe alterations in bony relationships [35,36]. A positional fault of the atlas could change normal movement in the upper cervical spine and mechanoreceptor activity, and cause abnormal muscle tone, resulting in pain with movement [35,36]. The concept of a positional default position of the atlas was further supported by the findings of Sillevis et al. [8], who demonstrated the presence of an asymmetrical position of the atlas on radiographs in subjects diagnosed with CGHs. The assumption that a positional default of the atlas would lead to a change in mobility was confirmed in this study. Post-intervention testing displayed a relative left-rotated positional default position of the atlas in 90% of the subjects. Additionally, there was a significant decrease in the right rotation of the atlas during the FRT. Since there was an inversed correlation between the post-intervention right OCI muscle length and the neck flexion right rotation range of motion, one can conclude that the increase in length of the right OCI muscle observed in this study seems responsible for the observed positional default and the reduced FRT right rotation mobility.

A method of direct muscle needling in combination with electrotherapy was utilized to isolate the left OCI and create a muscle contraction. This needle protocol was similar to Fernandez et al. [37], who demonstrated that 40 mm was long enough to reach the OCI. Penetrating needles through tissues can have a direct effect on such tissues. One of the proposed effects of dry needling directly into a muscle is a reduction of muscle hypertonicity [38]. The findings of our study do not support this, as the OCI demonstrated increased tone in the needled muscle. However, one could hypothesize that the induced muscle contractions utilizing electrotherapy had a more significant effect than the needling. Additionally, Rodrigues-Jiminez et al. [39] demonstrated that a single session of dry needling in the upper trapezius had a limited effect on outcome measures. This is supported by Funk et al. [40], who questioned if a single session of dry needling would have any significant clinical effect. This contrasts with the findings of Murillo et al. [41], who suggested that a single session of OCI needling increases upper cervical mobility. Murillo’s finding concurs with the result of this study, in which an overall change in motion at the atlantoaxial joint was demonstrated. Based on our study methodology, what cannot be discerned is the question: “Are the observed muscular changes in the OCI the result of needling versus induced tetanic muscle contraction?” Future studies should evaluate the needle effect of the OCI.

This study used MSK US and direct palpation to determine the positional relationship between the atlas and the axis. MSK US remains an emerging technology that allows practitioners to visualize anatomical structures in real time. Studies have shown that MSK US can reliably identify relevant structures in the upper cervical spine [3]. MSK US offers a real-time evaluation of the anatomy and has previously been used to determine the atlas and axis positional relationship [3]. MSK US has been shown to identify anatomical structures in the upper cervical spine with great accuracy [3,41]. Ashir et al. [42,43] reported that MSK US can be used to measure both muscle thickness and length reliably. The accuracy and reliability of MSK US in imaging the OCI have also been established [7]. With practice, the researchers in this study were able to create reliable and consistent images of the OCI [44,45]. The results of this study provide evidence that a change in muscle length of the right OCI muscle was visualized using MSK US. This implies that the distance between the right transverse process of the atlas and the axis spinous process had increased (*p* > 0.001). Therefore, it can be inferred that a left rotation of the atlas in the atlantoaxial joint occurred immediately after the intervention.

The second method in this study to obtain an impression of the position of the atlas was direct palpation while the subject was seated. The bilateral transverse processes of the atlas were palpated, and an impression was obtained of the position in space relative to the head and shoulders in either neutral, right, or left rotations. Ferreira et al. [46] demonstrated that palpation of the transverse process is an accurate method. Pei-Feng et al. [47] cautioned, however, using palpation of the transverse process of the atlas as morphology and asymmetries are common. Palpation was not used to obtain an impression of the size and or symmetry in this study, but just an impression on the relative position in space.

The results of this study provide evidence that after two tetanic contractions, the left OCI muscle the atlas assumes a left positional default position. This study does not give much insight into how long this dysfunctional positional default remains in healthy subjects. Despite this, our findings are clinically meaningful and can assist clinicians in managing upper cervical dysfunction and cervicogenic headaches. Direct intra-muscular dry needling with a combination of electrical stimulation and dry needling can alter a positional default of the atlas position and thus change the biomechanics of the upper cervical spine. Clinically, palpation should be utilized to determine the position of the atlas as part of evaluating the upper cervical spine. Additionally, atlas position can be used to determine the effects of clinical applications, such as exercise and or manual therapy targeting the upper cervical spine.

### Limitations

The number of subjects completing this study was 31, with a maximum age of 41. This might limit the generalizability. MSK US imaging is a real-time modality that allows visualization of anatomical structures with great precision; however, the image depends on the sonographer’s skill and quality. The researchers had limited training in imaging the upper cervical spine. Evidence has shown that novice practitioners trained in MSK US can achieve the same diagnostic accuracy as highly experienced sonographers [23,24,48]. Filippucci et al. [48] demonstrated that a novice could obtain quality images after a relatively short two-hour training by an experienced sonographer and 24 non-consecutive hours of active scanning. Based on extended practice and a physical therapist’s training and knowledge in anatomical and biomechanical functioning of the cervical spine, reliable MSK US images should have been obtained in this study.

## 5. Conclusions

This study aimed to determine the effect of two 5-s tetanic contractions delivered through intramuscular dry-needling electric stimulation on the atlantoaxial joint’s mobility and the atlas position. The right OCI was visualized using MSK US. Using MSK US for OCI visualization is a reliable and valid method. Direct palpation of the bilateral transverse processes of the atlas was used to obtain an impression of the atlas position as either neutral, right, or left rotated. This study’s results support that palpation of the atlas significantly correlates with OCI muscle length as measured with MSK US. The length of the right OCI significantly increased (mean change of 4 mm) after left OCI electric stimulation induced two tetanic muscle contractions. An increase in the right OCI can only occur if the atlas turns left. Palpation for the position of the atlas confirmed this change immediately after inducing the contractions. A left positional default of the atlas was identified with palpation. This change in the atlas position correlated with a decreased motion found in the FRT. This study was novel in its attempt to isolate the OCI muscle, create muscle contractions, and evaluate the effect on the position and rotational mobility of the atlas. This study has immediate clinical applicability since the position of the atlas directly impacts the functioning of the upper cervical spine. Clinicians should evaluate patients with upper cervical spine related conditions such as CGHs for atlas position. Additionally, future researchers should continue to investigate the effects of single OCI muscle contraction, pathological tone, and or shortening in subjects with upper cervical dysfunction and pathology such as CGHs.

## Figures and Tables

**Table 1:** One-Sample Paired *t*-test of the pre- and post-length measure of the right OCI and the effect size.

**One-Sample Test**							
	**Test Value = 0**						
	**t**	**df**	**Significance**		**Mean Difference**	**95% Confidence Interval of the Difference**	
			**One-Sided *p***	**Two-Sided *p***		**Lower**	**Upper**
Post muscle length	45.563	31	<0.001	<0.001	4.08656	3.9036	4.2695
Pre muscle length	38.364	31	<0.001	<0.001	4.06406	3.8480	4.2801
**One-Sample Effect Sizes**							
			**Standardizer**		**Point Estimate**	**95% Confidence Interval**	
						**Lower**	**Upper**
Pre muscle length	Cohen’s d		0.50737		8.054	6.026	10.075
	Hedges’ correction		0.52007		7.858	5.879	9.829
Post muscle length	Cohen’s d		0.59925		6.782	5.063	8.493
	Hedges’ correction		0.61425		6.616	4.940	8.285

**Table 2:** Data results for a Pearson’s correlation test between the diameter of the right OCI post-test and the range of motion of left and right rotation using the flexion–rotation test.

		Post Muscle Length	Post Flex Rotation Right	Post Flex Rotation Left
Post muscle length	Pearson’s correlation	1	−0.405 *	0.038
	Sig. (2-tailed)		0.022	0.835
	N	32	32	32
Post flex rotation right	Pearson’s correlation	−0.405 *	1	0.475 **
	Sig. (2-tailed)	0.022		0.006
	N	32	32	32
Post flex rotation left	Pearson’s correlation	0.038	0.475 **	1
	Sig. (2-tailed)	0.835	0.006	
	N	32	32	32

* Correlation is significant at the 0.05 level (2-tailed). ** Correlation is significant at the 0.01 level (2-tailed).

## Data Availability

The original contributions presented in this study are included in the article, further inquiries can be directed to the corresponding author.
